# Enhancing Compliance Through Local Anesthesia in Extended-Release Buprenorphine Therapy: A Report of Two Cases

**DOI:** 10.7759/cureus.109947

**Published:** 2026-05-30

**Authors:** Sina Radparvar

**Affiliations:** 1 Addiction Medicine, Kaiser Permanente West Los Angeles Medical Center, Sylmar, USA

**Keywords:** extended-release buprenorphine, injection-site pain, lidocaine, local anesthesia, opioid use disorder

## Abstract

In recent years, treatment options for opioid use disorder have expanded significantly with the availability of a wide range of medication formulations. Extended-release buprenorphine is a long-acting, injectable medication used to treat moderate-to-severe opioid use disorder by providing a steady, continuous dose of buprenorphine over several weeks. Needle-related anxiety and injection-site pain are frequent adverse events that can significantly impede patient adherence to extended-release buprenorphine therapy. Topical and injectable anesthetics can enhance patient comfort and support greater adherence to the treatment protocol.

This report presents two cases detailing a protocol to minimize pain from extended-release buprenorphine injections. This protocol utilizes a combination of topical anesthetic skin refrigerant (vapocoolant spray), local anesthetic, and an alkalizing agent, using specific techniques to minimize discomfort. After achieving adequate local anesthesia, extended-release buprenorphine therapy was well-tolerated, with participants reporting an essentially painless procedure.

Topical and injectable anesthetics can improve patient comfort during extended buprenorphine administration, boosting adherence to opioid maintenance therapy.

## Introduction

Opioid use disorder (OUD) poses a growing threat to global public health. In the United States, the opioid epidemic has transformed into a major public health crisis, resulting in over 700,000 deaths in the last two decades [1]. The age-adjusted rate of drug overdose deaths increased from 8.9 deaths per 100,000 standard population in 2003 to 32.6 in 2022; however, the rate decreased to 31.3 in 2023 [2].

Opioid agonist therapy (OAT), which includes methadone and buprenorphine, is an effective treatment for OUD. OAT has been shown to improve treatment retention, reduce unregulated opioid use, and reduce mortality risk [3]. However, the risk of death rises sharply after treatment discontinuation, underscoring the importance of medication adherence and treatment retention [3]. Oral OAT formulations pose challenges of relapse and poor adherence. Over the last decade, two distinct injectable subcutaneous extended-release buprenorphine (BUP-XR) formulations have been developed. Sublocade utilizes Atrigel technology, injecting a liquid polymer that solidifies into a solid, biodegradable depot for controlled monthly release [4]. Conversely, Brixadi and Buvidal utilize FluidCrystal technology, which transforms a low-viscosity lipid-based liquid into a biodegradable liquid crystalline gel matrix to provide sustained release [4].

Injectable BUP-XR is administered subcutaneously in the abdomen, thigh, buttock, or back of the upper arm once weekly or monthly, eliminating the need for daily dosing [4, 5]. Subcutaneous BUP-XR injections alleviate the cognitive burden of daily dosing, ensure stable plasma levels, and reduce the risk of pediatric accidental exposure [6]. This approach mitigates stigma associated with pharmacy pickups or methadone clinics and eliminates risks of lost, stolen, or diverted medication, supporting its logical use in recovery.

Patients transitioning from transmucosal buprenorphine can start BUP-XR via a rapid initiation protocol. This requires only a single transmucosal dose followed by a one-hour observation period to confirm tolerability [5]. Additionally, micro induction allows buprenorphine to be co-administered with full agonists, further lowering the barrier to entry for patients. 

Despite the less frequent administration, recent evidence has failed to show that extended-release injectable formulations are associated with improved adherence or cost-efficiency when compared with oral formulations [3]. Injection site pain during administration may impact patient comfort, adherence, and outcome. 

In 2019, a multicenter, randomized, double-blind, placebo-controlled, phase 3 trial included 404 participants with moderate to severe opioid use disorder who received BUP-XR [7]. Side effects included injection-site pruritus (32 (8%) of 404 vs. 4 (4%) of 100) and injection-site pain (22 (5%) of 404 vs. 3 (3%) of 100) in the active and placebo groups, respectively [7].

In 2020, a 12-month multicenter phase 3 study of 669 adults with moderate to severe OUD, injection site adverse effects were reported by 13.2% of participants, with the most commong most common pain (6.9%), erythema (4.0 %), and pruritus (3.9%) [8].

Long-acting buprenorphine (BUP-XR) formulations differ in injection volume and needle size. Sublocade uses a larger 19-gauge needle and higher volumes (0.5-1.5 mL) [9], whereas Brixadi and Buvidal utilize smaller 23-gauge needles with lower volumes (0.16-0.64 ml) [10]. Compared to Brixadi or Buvidal, Sublocade is associated with greater patient discomfort, necessitating more frequent use of local anesthesia. Nonetheless, a local anesthetic may still be indicated for sensitive patients receiving Brixadi or Buvidal.

Proposed local anesthesia protocol prior to BUP-XR therapy

Regional anesthesia prior to BUP-XR administration may be a feasible option to minimize the needle-related anxiety and injection-site pain experienced by patients. Use of both topical and injectable anesthesia has been described in several protocols for BUP-XR injections [11, 12]. However, to the best of the author's knowledge, detailed published protocols are not available.

Effective protocols depend on understanding the nociceptive and pain mechanisms at the injection site. Pain transmission relies on a coordinated network where specialized neurons detect stimuli and relay them to the central nervous system. A-δ fibers convey fast, sharp pain; C fibers convey slow, dull pain. The dorsal root ganglia (DRG) house the cell bodies of these neurons and regulate signal flow. Nociceptors (pain sensors) detect harmful stimuli, which are transmitted via A-δ and C nerve fibers to the DRG [13]. DRG, which contains first-order pain afferents, plays an important role in conveying peripheral nociceptive information to the central nervous system (CNS) [14]. DRG neurons are characterized by the presence of voltage-gated sodium, potassium, and calcium channels, which modulate excitability and synaptic transmission [14].

Lidocaine is an amide-class anesthetic [15] that produces its effect by blocking neuronal sodium channels, regulating potassium currents, and modulating cellular calcium concentrations via ligand-gated channels [14]. Lidocaine works by blocking free nerve endings and preventing the transmission of painful stimuli [16]. It is the drug of choice for a variety of medical procedures due to its strong anesthetic potential, fast onset of action (two to five minutes), short duration (one to two hours), and wide safety limits [17]. Various methods can mitigate discomfort during BUP-XR administration, including topical anesthetic application prior to lidocaine injection, use of buffered lidocaine solutions, a smaller-diameter needle for infiltration, and refined injection techniques such as altering injection depth and angle and rate of anesthetic injection [18].

Topical Anesthesia

Before injected Lidocaine molecules can provide anesthesia, they must first diffuse through hydrophobic cell membranes into the neurons. Pain from local anesthetic injections occurs with both the insertion of the needle and the infiltration of fluid [16]. Topical anesthetic gels and vapocoolants have been used to decrease pain associated with needle insertion. Compared to topical gels, refrigerants provide faster pain relief and a more efficient application process [19].

Vapocoolants provide rapid topical anesthesia through evaporative cooling. Typically composed of low-boiling-point volatile mixtures, these agents evaporate immediately upon release, extracting heat from the skin. This sharp temperature drop disrupts nerve conduction in A-δ and C fibers, temporarily blocking pain signals to the brain. A 2-3 second application from a distance of 4-6 inches induces sufficient analgesia for painless injections or minor superficial procedures [20].

Buffered Lidocaine Solutions

Lidocaine is the standard office anesthetic, though its acidity typically causes a burning sensation during injection. Clinical studies demonstrate that buffering lidocaine with 8.4% sodium bicarbonate at a 3:1 ratio significantly reduces the pain of infiltration [21].

Needle Diameter 

Research indicates that a larger outer needle diameter is significantly linked to increased insertion pain [16, 22]. Using thinner needles, such as 27- to 30-gauge, reduces pain frequency and slows the injection rate, though the latter may slightly diminish the anesthetic's overall effect [16, 22]. Consequently, the 19-gauge needles required for Sublocade (BUP-XR) [9] and 23-gauge needles for Brixadi or Buvidal [10] likely cause greater discomfort without local anesthetic.

Injection Technique

Various techniques for Lidocaine administration have been published with the goal of minimizing the pain of local anesthetic injections. Several published protocols have recommended a multi-step process for local anesthetic injection [16, 23].

Before needle insertion, the non-dominant hand can be used to firmly pinch the skin proximal to the site, providing tactile distraction [23]. The rapid, firm pinching activates large-diameter, fast-conducting sensory nerve fibers (A-ß fibers) which transmit signals faster than smaller pain-sensing C fibers triggered by the needle [24]. The sensory input stimulates interneurons in the spinal cord's dorsal horn, which inhibits the transmission of nociceptive signals (A-δ and C fibers), thus reducing perceived injection pain [24].

The present case reports utilize the parallel, minimal-insertion technique, characterized by subdermal needle placement parallel to the skin surface to facilitate gradual anesthetic infiltration before reaching deeper layers [25]. This method has shown to decrease pain compared to the perpendicular injections [25]. Following intradermal anesthesia induction, the needle is advanced into the subcutaneous tissue to deliver more anesthetic solution utilizing pivoting or redirecting technique [25]. Finally, the needle is removed from the subcutaneous tissue using a retrograde infiltrative technique.

## Case presentation

Case one

A 34-year-old male presented to an outpatient addiction medicine clinic requesting help for "quitting heroin". He reported a five-year history of opioid use, which began with prescribed oxycodone for chronic back pain. Once prescriptions were discontinued, he switched to "street pills" and subsequently to intravenous heroin three years ago. He reported using approximately 0.5 to 1 gram of heroin daily, noting intense cravings and recurrent debilitating withdrawal symptoms. Social history revealed that the patient was divorced, unemployed, and lived in shared housing, having lost his job as a software developer due to absenteeism and declining performance. Upon initial assessment, the patient appeared anxious with poor personal hygiene. Heart rate was 102 beats per minute, blood pressure was 135/95, and respiratory rate was 16 breaths per minute. The patient exhibited restlessness, frequent yawning, and reported chills and muscle aches. Examination showed multiple "track marks" and scarring (injection site necrosis) in the antecubital fossae.

Laboratory testing revealed the patient is positive for hepatitis C (HCV). Testing was negative for HIV. Urine toxicology was positive for morphine and 6-monoacetylmorphine (6-MAM). The patient was diagnosed with severe opioid use disorder based on Diagnostic and Statistical Manual of Mental Disorders, 5th Edition (DSM-5) criteria [26]. He also screened positive for generalized anxiety disorder and major depressive disorder, which he reported worsened after his divorce. 

The patient's symptoms were assessed using the Clinical Opioid Withdrawal Scale (COWS), which is an 11-item clinician-administered tool used to reproducibly rate the severity of opioid withdrawal signs and symptoms [27]. Assessment criteria include physiological indicators such as resting pulse rate, pupil size, tremors, and the presence of gooseflesh skin, as well as observable behaviors like restlessness and yawning. Clinicians also evaluate patient-reported symptoms, including gastrointestinal upset, anxiety or irritability, bone or joint aches, and a runny nose or tearing. Total scores range from 0 to 48, with severity categorized as mild (5-12), moderate (13-24), moderately severe (25-36), or severe (greater than 36) [27]. Assessment of the current patient indicated moderate withdrawal severity (COWS 14).

The treatment plan included a multidisciplinary approach focusing on medication-assisted treatment (MAT) and psychological support. Following induction with a 4 mg sublingual dose of buprenorphine/naloxone, the right upper abdominal skin, specifically the area between the transpyloric and transtubercular planes, was prepared. The patient was informed of the procedure for the induction of local skin anesthesia followed by subcutaneous medication injection. The patient initially expressed apprehension but tolerated the discussion well after receiving reassurance and counseling. Procedural anesthesia was achieved using a combination of a vapocoolant spray (pentafluoropropane/tetrafluoroethane) and 1% lidocaine with 8.4% sodium bicarbonate, according to the protocol in Table 1, which outlines the local anesthesia procedure for extended-release buprenorphine injection. Sublocade 300 mg was injected subcutaneously 10 minutes after the administration of anesthesia. The patient tolerated the procedure and reported a 2 out of 10 on the numeric pain scale, describing the pain as mild during the procedure. No procedural adverse events such as burning, redness, or allergic response occurred. At five minutes post‑injection, the patient reported no recall of significant pain. During subsequent injections, pain scores from previous procedures were reviewed to determine if protocol adjustments (such as increasing anesthetic volume) were indicated; however, no such changes were required. The procedure was well-tolerated with no local or systemic complications, including burning or allergic response. The patient was referred for cognitive behavioral therapy (CBT) to address underlying depression and develop coping strategies for cravings. Referrals were made to a hepatologist for hepatitis C management and a primary care physician for non-opioid chronic pain management. He was also provided with a naloxone (Narcan) rescue kit and education on overdose prevention. He was seen monthly for BUP-XR injection and was able to maintain sobriety for six months thereafter.

**Table 1:** Local anesthetic administration technique before extended-release buprenorphine injection

Step	Action	Parameters
1	Lidocaine injection preparation	Aseptically withdraw 4 ml 1% lidocaine injection. Mix with 1.3 ml 8.4% sodium bicarbonate (3:1 ratio), followed by application of a 27-30 gauge needle.
2	Topical antiseptic	Prepare the site with an alcohol-based antiseptic, moving from the center outward or using a back-and-forth scrub. Allow the air to dry completely.
3	Vapocoolant spray (hydrofluorocarbons)	Spray the vapocoolant for a duration of 2-3 seconds, maintaining a distance of 4-6 inches from the injection site.
4	Tactile distraction	Utilize tactile stimulation by applying gentle pressure to the area adjacent to the injection site for 5 seconds.
5	Cognitive distraction	Utilize visual distraction by instructing the patient to look away to mitigate anxiety, fear, and pain perception during needle insertion.
6	Needle insertion & injection technique	Intradermal anesthesia induction: Establish an initial anesthetic wheal by injecting 0.5 ml of anesthetic solution into the superficial dermis at a 10–15 degree angle (parallel minimal insertion technique). Wait 30-60 seconds for the anesthetic to take effect and numb the area. Deep tissue targeting via needle manipulation: With the needle tip in the sub-dermal layer, guide it toward deeper tissue by withdrawing almost to the entry point without exiting the skin and redirecting at a new horizontal angle. Maintain slow, steady injection pressure while advancing to keep the tip bathed in anesthesia. Advance the needle through the dermis into the subcutaneous tissue, injecting 2.5 ml of anesthetic fluid. Retrograde infiltration: Slowly withdraw the needle from the subcutaneous tissue, injecting the remaining 2.3 ml of anesthetic solution during the process.
7	Injection pain score	Evaluate the patient's procedural pain intensity using a 0-10 numeric rating.
8	Wait Time and Testing	After a 5–10 minute wait, confirm adequate anesthesia via the pinprick test before administering the extended-release buprenorphine.

Case two

A 52-year-old female presented to the Addiction Medicine clinic reporting use of "natural supplements" to self-treat fibromyalgia, stating "I can't function without it and feel sick if I stop". The patient reported three-year dependency on kratom (*Mitragyna speciosa*), which she initially discovered online as a "non-addictive" alternative to prescription opioids for managing fibromyalgia and chronic hip pain. Over the last year, use escalated to consuming 35-40 grams of raw leaf powder daily. The patient reported that the initial therapeutic benefit (energy boost, pain relief) subsided. Current usage was primarily to avoid withdrawal symptoms, which included severe anxiety, restless legs, and diaphoresis occurring six hours post-dose. She also complained of chronic constipation.

Social history was notable for the patient being married, living with her husband, and working as an administrative assistant.

Medical history was significant for fibromyalgia, chronic hip osteoarthritis, and a history of iron-deficiency anemia. Psychiatric history was significant for depression. The patient reported initial mood improvement with kratom, followed by emergent irritability and cognitive dysfunction ('brain fog'). Patient denied alcohol, tobacco, or other drug use.

Physical examination showed heart rate 94 beats per minute, blood pressure 138/88 mmHg, respiratory rate 15, and temperature 99.1°F. The skin had notable hyperpigmentation on the cheeks and bridge of the nose. COWS score was 8 (early to mild withdrawal). Laboratory testing showed elevated liver enzymes with potential for kratom-induced liver injury. Initial diagnosis showed severe opioid use disorder based on DSM-5 criteria [26].

The treatment plan included a discussion of a gradual kratom taper with subsequent transition into the extended-release Buprenorphine. The patient was prescribed clonidine for autonomic dysfunction, gabapentin for restless legs, and acetaminophen for pain.

The patient was reassessed 72 hours after the initial evaluation, at which time 4 mg of sublingual buprenorphine was administered. The patient tolerated the dose well, with no signs of precipitated withdrawal or adverse effects observed. After a one-hour observation, the patient's left lower abdominal skin area between the transpyloric and transtubercular planes was prepared. Patient acknowledged past history of needle anxiety. Following a detailed discussion of the procedure to provide reassurance, the patient appeared calm and provided verbal consent to proceed. Procedural anesthesia was achieved using a combination of a vapocoolant spray (pentafluoropropane/tetrafluoroethane) and 1% lidocaine with 8.4% sodium bicarbonate, according to the protocol in Table 1. After 10 minutes. Brixadi 16 mg was injected subcutaneously. Brixadi 8 mg injections were subsequently administered on days four and six, with no adverse reactions reported. Patient tolerated the procedure with minimal discomfort, reporting 1 out of 10 on the numeric pain scale. No procedural adverse events occurred. At five minutes post‑injection, the patient reported no recall of significant pain. Brixadi was selected for the initial week to utilize its dose flexibility, enabling faster stabilization during early withdrawal, making it an ideal choice for tapering patients off kratom. The patient was subsequently transitioned to Sublocade 300 mg subcutaneously monthly. All injections were administered in accordance with the protocol in Table 1 and were well tolerated, with patients reporting 1-2 out of 10 on the numerical pain scale. During subsequent injections, pain scores from previous procedures were reviewed to determine if protocol adjustments (such as increasing anesthetic volume) were indicated; however, no such changes were required. The procedure was well-tolerated with no local or systemic complications, including burning or allergic response. The patient was referred to an addiction-informed therapist to address the "natural" supplement misconception and develop new coping strategies for chronic pain. She was also referred to a primary care physician for treatment of fibromyalgia. One month later, the patient was seen for BUP-NX injection. Repeat liver function tests were normal.

Psychiatric assessment showed major depression, and she was started on Cymbalta 60 mg daily. She was able to maintain long-term sobriety while on monthly Sublocade maintenance.

**Figure 1:**
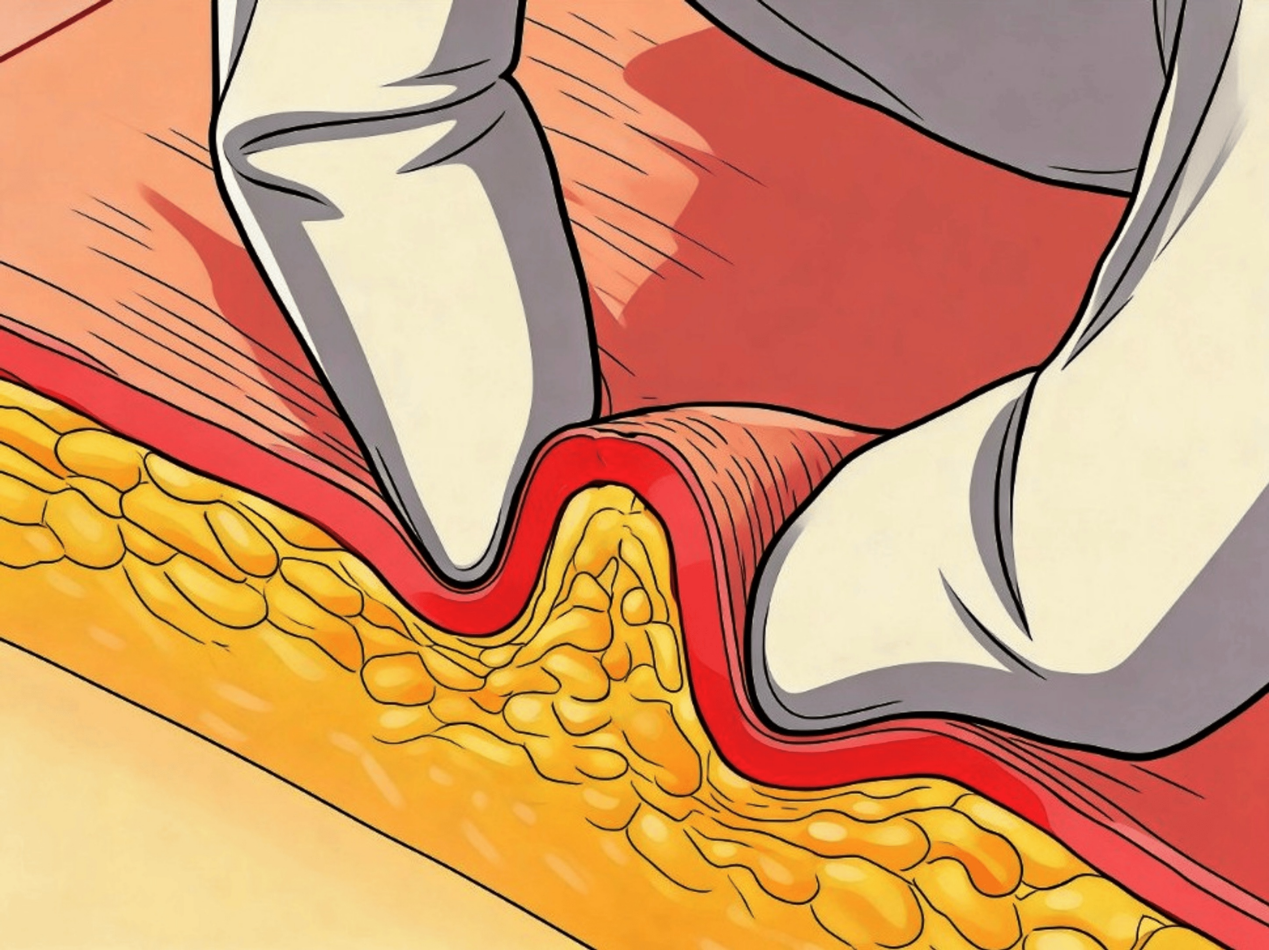
Utilizing the gate control theory, tactile stimulation (pinching/wiggling) proximal to the injection site activates mechanoreceptors, inhibiting nociceptive input from needle insertion Image was created by the authors using GIMP (GIMP, San Francisco, California).

**Figure 2:**
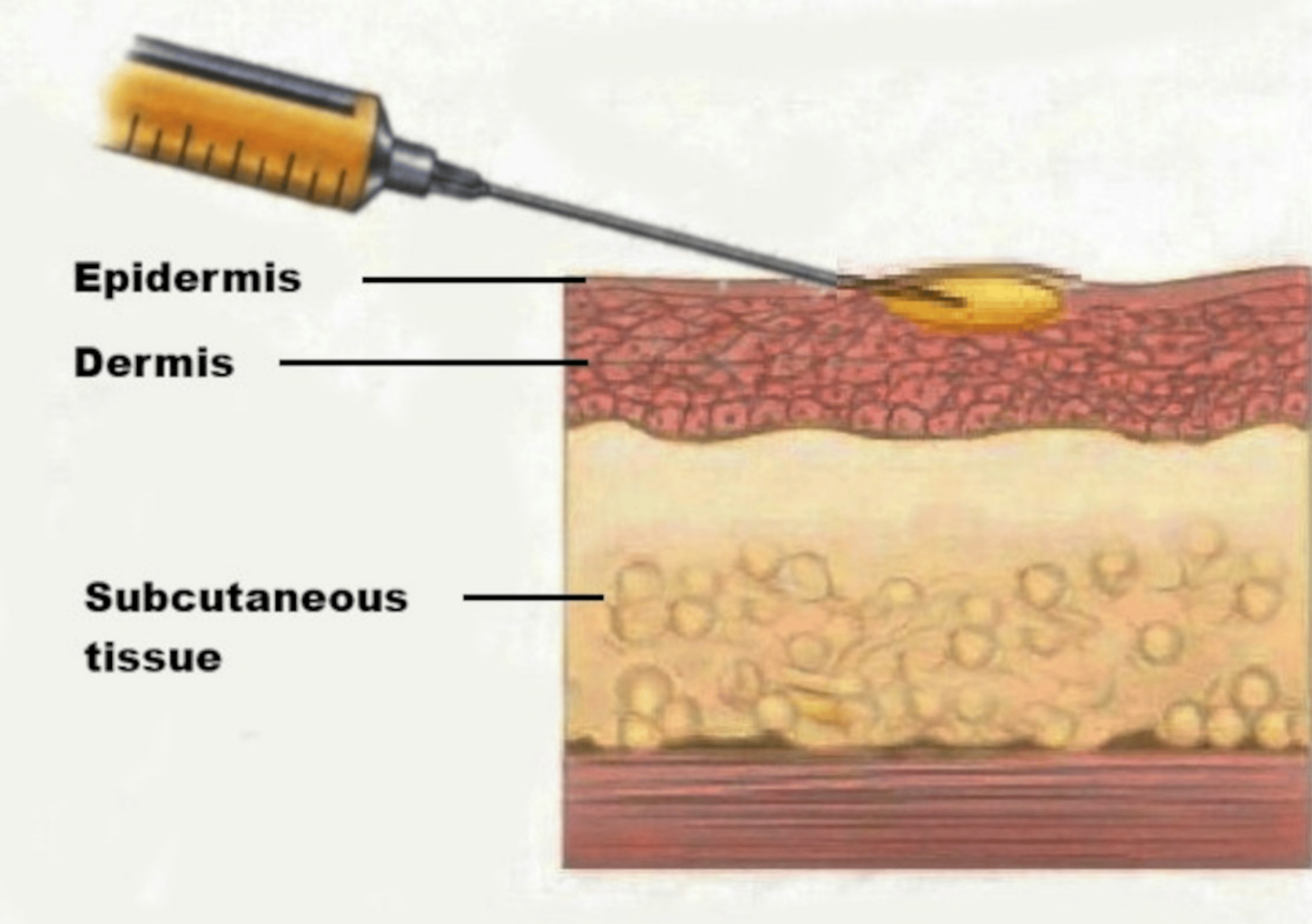
Intradermal injection of 0.5 ml anesthetic, using a 25–27 gauge needle at a 10–15° angle (bevel down), creates a 3–5 mm blanched wheal to provide superficial anesthesia before deeper infiltration Image was created by the authors using GIMP (GIMP, San Francisco, California).

**Figure 3:**
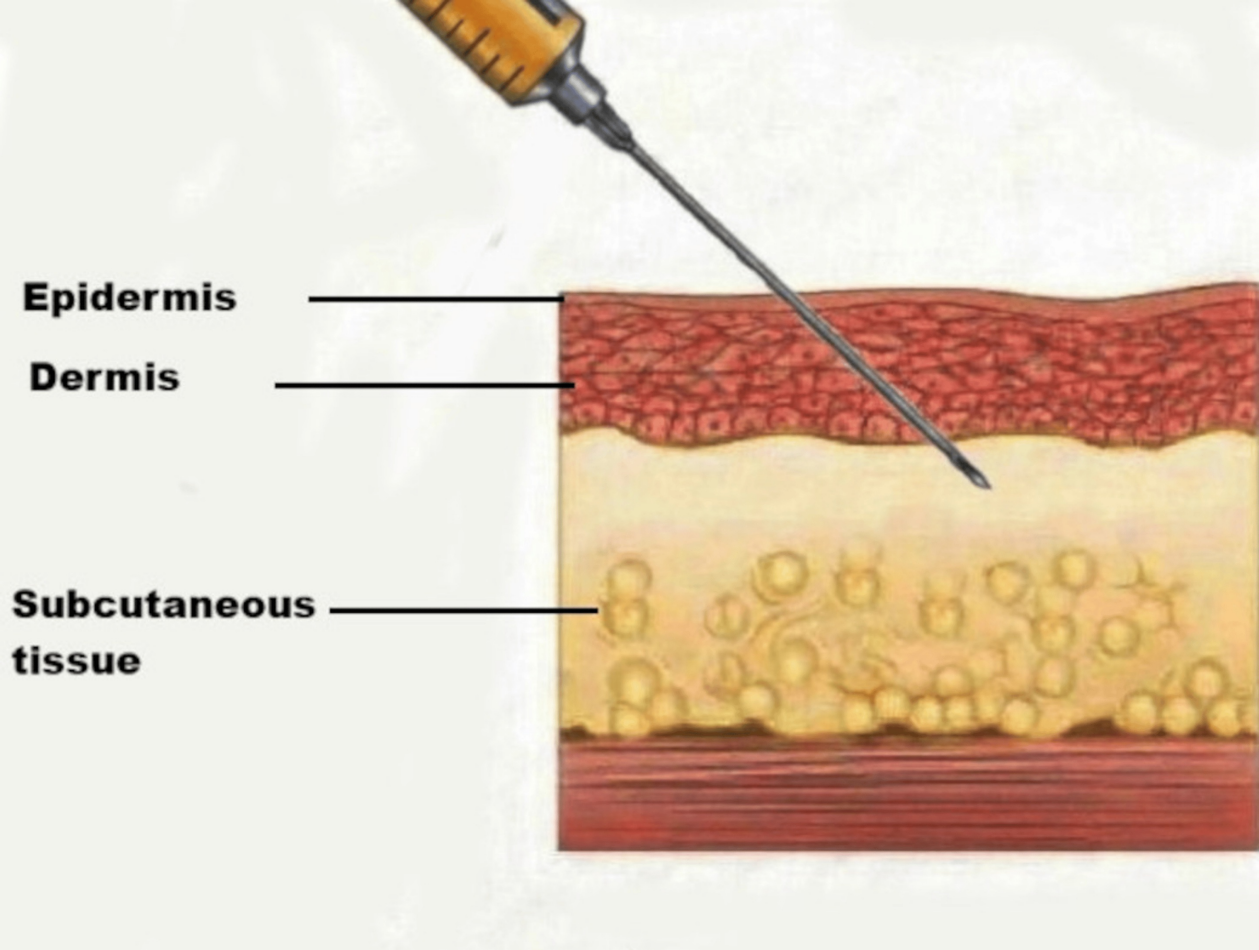
The needle tip is redirected through the dermis at a deeper horizontal angle to reach the subcutaneous tissue without a secondary puncture Image was created by the authors using GIMP (GIMP, San Francisco, California).

**Figure 4:**
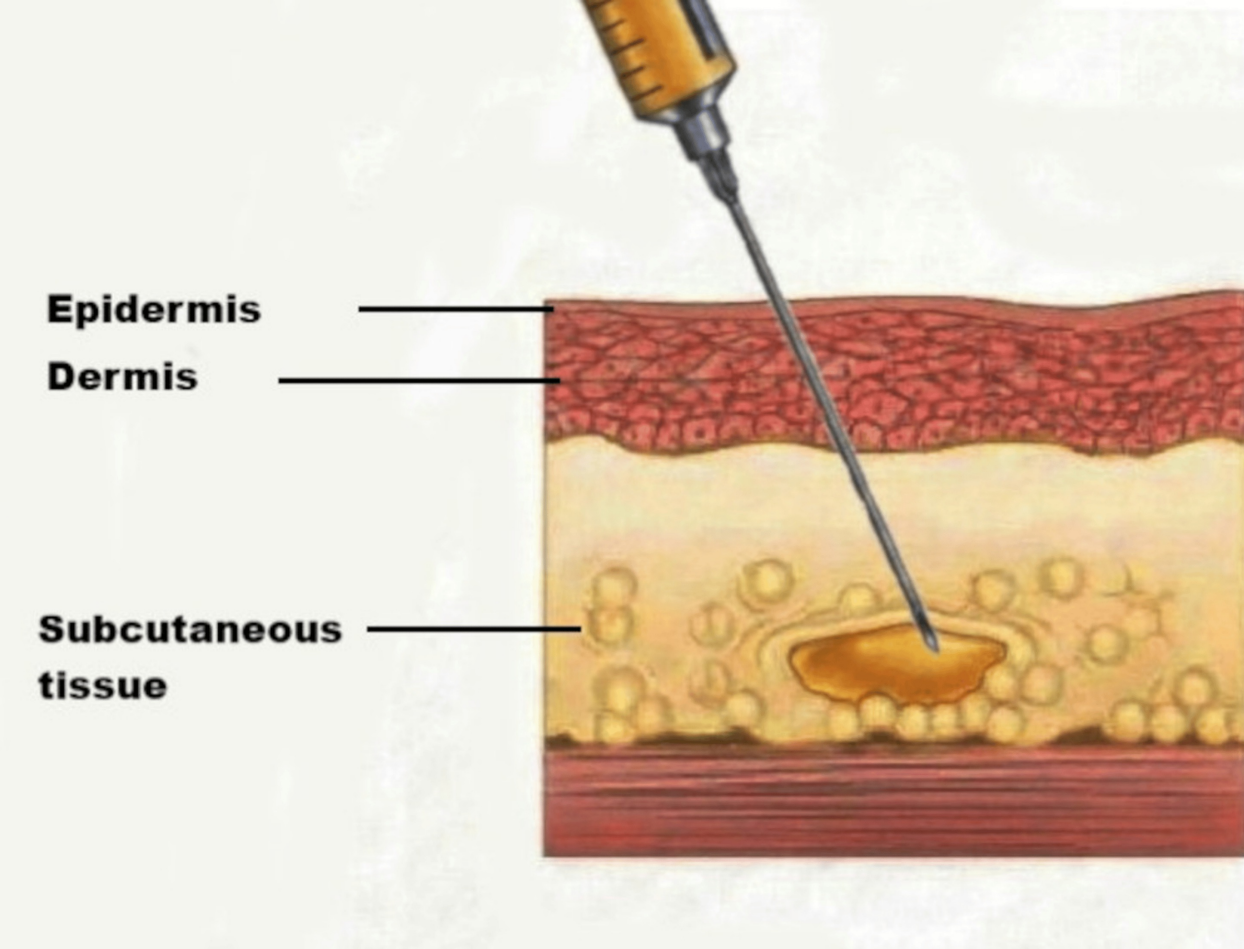
The needle tip is further redirected and advanced deeper into the subcutaneous tissue, while maintaining a constant, slow injection rate of roughly 0.3 ml per second Image was created by the authors using GIMP (GIMP, San Francisco, California).

The injection technique described in the protocol is illustrated in Figures 1-4.

## Discussion

OAT with buprenorphine or methadone is well established as effective treatment for opioid use disorder. By interrupting the cycle of intoxication and withdrawal, OAT greatly reduces illicit opioid use, risk of overdose and mortality. However, despite the efficacy of OAT, treatment attrition is high. Retaining patients with opioid use disorder on OAT is challenging as relapse or dropout affects nearly half of patients (40-50% retention rates) within a 6 month period of treatment initiation with buprenorphine or methadone [28].

Despite expectations that BUP-XR would improve adherence, real-world data shows it underperforms oral formulations, exhibiting lower retention rates [29].

A study published in 2022, investigated retrospective chart review (observational cohort study) assessing treatment retention in a primary care setting for adults with opioid use disorder (OUD) transitioning from daily sublingual to monthly injectable BUP-XR. It found high early discontinuation, with 48% of patients discontinuing BUP-XR within 3 months [6].

A 2024 follow-up to the CoLAB study (a prospective, single-arm, multicenter, open-label trial) found that only 47% of participants remained in treatment with BUP-XR at 96 weeks [30]. This study evaluated long-term retention of extended-release subcutaneous buprenorphine among people with opioid dependence [30]. An observational score-matched cohort study published in 2025 found that patients switching to subcutaneous BUP-XR had lower treatment retention (median 269 days) compared to those on the sublingual formulation (389 days) [31].

Low retention rates for extended-release injectable buprenorphine are linked to several, often overlapping, factors. Key barriers include high medication costs, requests for prior authorization and limited pharmacy access [29, 32]. Inadequate care coordination during the transition from daily sublingual to monthly injectable and provider experience are also significant factors affecting patient retention rates [29, 33]. Additionally, patient-related factors such as perceptions, experiences, and satisfaction are also important. In several studies, patients have reported challenges to therapy focused on fear of needles, pain and temporary injected site discomfort [8, 34-35]. Further research is needed to investigate patient experience and comfort during BUP-XR administration, focusing on reducing procedural pain and needle phobia, along with strategies to mitigate these barriers.

## Conclusions

Long-acting injectable buprenorphine therapy has the potential to revolutionize OUD treatment by enhancing patient quality of life, reducing the stigma of daily medication dosing, and improving medication adherence. Negative effects such as injection site pain are typically transient; however, they can result in treatment discontinuation/non-adherence. The anesthetic protocol detailed in this report demonstrates the potential to mitigate patient anxiety and enhance comfort during the procedure. While both patients reported no pain under this protocol, further controlled studies are necessary to confirm whether the use of local anesthetics before buprenorphine injection can improve patient comfort, boost engagement, enhance long-term retention, and promote sustained recovery in patients with opioid use disorder. 

## References

[1] Saunders H, Panchal N, Rudowitz R (2026). Opioid overdose deaths: National trends and variation by demographics and states. https://www.kff.org/mental-health/opioid-overdose-deaths-national-trends-and-variation-by-demographics-and-states/.

[2] Garnett MF, Miniño AM (2026). Drug overdose deaths in the United States, 2003-2023. https://www.cdc.gov/nchs/data/databriefs/db522.pdf.

[3] Siu JT, Lau A, Wong JS, Machado J, Azar P (2025). Transition to extended-release buprenorphine injectable within seven days for opioid use disorder treatment: a scoping narrative. J Addict Med.

[4] Ling W, Shoptaw S, Goodman-Meza D (2019). Depot buprenorphine injection in the management of opioid use disorder: from development to implementation. Subst Abuse Rehabil.

[5] (2026). Indivior announces FDA approval of label changes for Sublocade (buprenorphine extended-release) injection. Indivior PLC..

[6] Stein MD, VanNoppen D, Herman DS, Anderson BJ, Conti M, Bailey GL (2022). Retention in care for persons with opioid use disorder transitioning from sublingual to injectable buprenorphine. J Subst Abuse Treat.

[7] Haight BR, Learned SM, Laffont CM (2019). Efficacy and safety of a monthly buprenorphine depot injection for opioid use disorder: a multicentre, randomised, double-blind, placebo-controlled, phase 3 trial. Lancet.

[8] Andorn AC, Haight BR, Shinde S (2020). Treating opioid use disorder with a monthly subcutaneous buprenorphine depot injection: 12-month safety, tolerability, and efficacy analysis. J Clin Psychopharmacol.

[9] (2026). Sublocade (buprenorphine) extended-release injection for subcutaneous use. https://www.accessdata.fda.gov/drugsatfda_docs/label/2023/209819s028lbl.pdf.

[10] (2026). Brixadi (buprenorphine) extended-release injection for subcutaneous use. https://www.brixadi.com/pdfs/brixadi-prescribing-information.pdf.

[11] Ganetsky VS (2026). Clinical updates on the use of injectable medications for opioid use disorder (MoUD). https://sites.rutgers.edu/mat-coe/wp-content/uploads/sites/473/2021/05/Clinical-Updates-on-the-Use-of-Injectable-Medications-for-Opioid-Use-Disorders-MOUD-05072021.pdf.

[12] Kan D, Mascorro A (2026). Using Sublocade in your MAT program. https://www.careinnovations.org/wp-content/uploads/Sublocade-Presentation_060820.pdf.

[13] Ahimsadasan N, Reddy V, Khan Suheb MZ, Kumar A (2026). Neuroanatomy, Dorsal Root Ganglion. StatPearls [Internet].

[14] Yang X, Wei X, Mu Y, Li Q, Liu J (2020). A review of the mechanism of the central analgesic effect of lidocaine. Medicine (Baltimore).

[15] Karnina R, Arif SK, Hatta M, Bukhari A (2021). Molecular mechanisms of lidocaine. Ann Med Surg (Lond).

[16] Strazar AR, Leynes PG, Lalonde DH (2013). Minimizing the pain of local anesthesia injection. Plast Reconstr Surg.

[17] Golzari SE, Soleimanpour H, Mahmoodpoor A, Safari S, Ala A (2014). Lidocaine and pain management in the emergency department: a review article. Anesth Pain Med.

[18] Zelickson BR, Goldberg LH, Rubenzik MK, Wu WJ (2018). Finer needles reduce pain associated with injection of local anesthetic using a minimal insertion injection technique. Dermatol Surg.

[19] Kosaraju A, Vandewalle KS (2009). A comparison of a refrigerant and a topical anesthetic gel as preinjection anesthetics: a clinical evaluation. J Am Dent Assoc.

[20] Plotkin S (1998). Clinical comparison of preinjection anesthetics. J Am Podiatr Med Assoc.

[21] Vent A, Surber C, Graf Johansen NT (2020). Buffered lidocaine 1%/epinephrine 1:100,000 with sodium bicarbonate (sodium hydrogen carbonate) in a 3:1 ratio is less painful than a 9:1 ratio: a double-blind, randomized, placebo-controlled, crossover trial. J Am Acad Dermatol.

[22] Arendt-Nielsen L, Egekvist H, Bjerring P (2006). Pain following controlled cutaneous insertion of needles with different diameters. Somatosens Mot Res.

[23] Joukhadar N, Lalonde D (2021). How to minimize the pain of local anesthetic injection for wide awake surgery. Plast Reconstr Surg Glob Open.

[24] Fosko SW, Gibney MD, Harrison B (1998). Repetitive pinching of the skin during lidocaine infiltration reduces patient discomfort. J Am Acad Dermatol.

[25] Ceilley RI, Sureshbabu S (2025). Optimizing local anesthesia use in office-based dermatologic procedures. J Clin Aesthet Dermatol.

[26] American Psychiatric Association (2026). Opioid-related disorders. Diagnostic and Statistical Manual of Mental Disorders.

[27] Wesson DR, Ling W (2003). The Clinical Opiate Withdrawal Scale (COWS). J Psychoactive Drugs.

[28] Compton WM, Volkow ND (2021). Extended-release buprenorphine and its evaluation with patient-reported outcomes. JAMA Netw Open.

[29] Ivasiy R, Madden LM, Johnson KA (2025). Retention and dropout from sublingual and extended-release buprenorphine treatment: a comparative analysis of data from a nationally representative sample of commercially-insured people with opioid use disorder in the United States. Int J Drug Policy.

[30] Farrell M, Shahbazi J, Chambers M (2024). 96-week retention in treatment with extended-release subcutaneous buprenorphine depot injections among people with opioid dependence: Extended follow-up after a single-arm trial. Int J Drug Policy.

[31] Deng C, Oviedo E, Fishman M, Burgess-Hull A (2025). A comparative study of treatment retention in opioid use disorder: Subcutaneous injectable versus sublingual buprenorphine. Addiction.

[32] Andraka-Christou B, Simon KI, Bradford WD, Nguyen T (2023). Buprenorphine treatment for opioid use disorder: comparison of insurance restrictions, 2017-21. Health Aff (Millwood).

[33] Reddy IA, Audet CM, Reese TJ, Peek G, Marcovitz D (2024). Provider perceptions toward extended-release buprenorphine for treatment of opioid use disorder. J Addict Med.

[34] Parsons G, Ragbir C, D'Agnone O, Gibbs A, Littlewood R, Hard B (2020). Patient-reported outcomes, experiences and satisfaction with weekly and monthly injectable prolonged-release buprenorphine. Subst Abuse Rehabil.

[35] Cheng A, Badolato R, Segoshi A (2022). Perceptions and experiences toward extended-release buprenorphine among persons leaving jail with opioid use disorders before and during COVID-19: an in-depth qualitative study. Addict Sci Clin Pract.

